# Notch4 is activated in endothelial and smooth muscle cells in human brain arteriovenous malformations

**DOI:** 10.1111/jcmm.12115

**Published:** 2013-11-05

**Authors:** Qichuan ZhuGe, Zhebao Wu, Lijie Huang, Bei Zhao, Ming Zhong, WeiMing Zheng, Chen GouRong, XiaoOu Mao, Lin Xie, Xiangdong Wang, Kunlin Jin

**Affiliations:** a Zhejiang Provincial Key Laboratory of Aging and Neurological Disorder Research First Affiliated Hospital, Wenzhou Medical UniversityWenzhou, China; b Institute of Clinical & Translational Science First Affiliated Hospital, Wenzhou Medical UniversityWenzhou, China; c Buck Institute for Research on AgingNovato, CA, USA; d Department of Pharmacology and Neuroscience, University of North Texas Health Science CenterFort Worth, TX, USA

**Keywords:** Notch4, AVM, human, brain, signalling

## Abstract

Up-regulation of Notch4 was observed in the endothelial cells in the arteriovenous malformations (AVMs) in mice. However, whether Notch4 is also involved in brain AVMs in humans remains unclear. Here, we performed immunohistochemistry on normal brain vascular tissue and surgically resected brain AVMs and found that Notch4 was up-regulated in the subset of abnormal vessels of the brain AVM nidus, compared with control brain vascular tissue. Two-photon confocal images show that Notch4 was expressed not only in the endothelial but also in the smooth muscle cells of the vascular wall in brain AVMs. Western blotting shows that Notch4 was activated in brain AVMs, but not in middle cerebral artery of normal human brain, which was confirmed by immunostaining. Our findings suggest a possible contribution of Notch4 signalling to the development of brain AVMs in human.

## Introduction

Brain arteriovenous malformations (AVMs) are morphological abnormalities characterized by direct communication between arteries and veins without intervening capillary beds. Brain AVMs are the single most common cause of stroke, which occurs as a result of intracerebral haemorrhage in young adults. Arteriovenous malformations are generally thought to be congenital vascular anomalies that arise as a result of the abnormal development of blood vessels during the early embryonic period 1. However, most brain AVMs are sporadic; therefore, the genetic clue leading to brain AVM remains undefined.

Notch1 signalling in link with AVMs was initially suggested based on the findings that Notch1 signalling is required for the proper development of arterial and venous blood vessels, as mice with defects in genes encoding Notch1 and components of the Notch signalling cascade invariably display vascular defects 2–3. Notch signalling-deficient embryos, such as embryos with an endothelial cell-specific deletion of the *Notch1* gene, exhibit a poorly formed dorsal aorta and posterior cardinal vein with accompanying AVMs 3–4. Targeted disruption of the *Jagged1, Notch1 ligand,* in mice also results in defects in head and yolk sac angiogenesis 5. We found that Notch1 signalling is activated in both endothelial and smooth muscle cells (SMC) of human brain AVMs. In addition, Hes1, downstream target of Notch signalling, is activated in human brain AVMs 6–7. Continuous intraventricular administration of a Notch1 activator in normal rats stimulates the proliferation of both endothelial and vascular SMCs, suggesting that enhanced Notch1 signalling can independently induce a pro-angiogenic state 6.

In addition to Notch1, studies show that Notch4 signalling also plays an important role in regulating vascular development. For example, the survival of Notch4-deficient mice shows that Notch4 is dispensable for vascular development 8, while expression of an activated form of Notch4 within the endothelium disrupts normal vascular development 9–10. A recent study shows that mice with constitutively active Notch4 (*int3*) expression in endothelial cells from birth develop hallmarks of brain AVMs, including cerebral arteriovenous shunting and vessel enlargement by 3 weeks of age 11. These results suggest that proper activation level of Notch signalling is critical for vascular development. The question that remains is whether Notch4 receptor is involved in human brain AVMs.

In this study, we analysed the expressions of Notch4 in the specimens of human brain AVMs. We found that Notch4 was expressed in the endothelial and the SMCs of the vascular wall in brain AVMs. Western blotting and confocal images show that Notch 4 was activated in brain AVMs. Our data suggest that activation of Notch signalling may play an important role in the pathogenesis of brain AVM in human.

## Material and methods

### Human brain specimens

Eight brain specimens from patients with brain AVMs were obtained from the First Affiliated Hospital at the Wenzhou Medical University by surgical resection between 2005 and 2007. The patients ranged in age from 10 to 67 years with a mean of 44.25 years (median 44). Patient information is summarized in Table 1. Four normal human brain specimens without clinical or postmortem evidence of neurological diseases were obtained from the Brain and Tissue Bank for Developmental Disorders of the National Institute of Child and Health and Human Development (University of Maryland, Baltimore, MD, USA). All studies involving patients were conducted under protocols approved by the Buck Institute for Research on Aging and Wenzhou Medical University, China.

**Table 1:** Summary of clinical information on patients with brain AVMs

Case no.	Age (yr)	Sex	Initial sign	Cerebral AVM	
				Location*	Diameter (cm)
1	30	M	Epilepsy	R P/O	3.50
2	36	M	Epilepsy	L T	2.66
3	10	M	Headache	L T	1.50
4	56	M	Haemorrhage	L F/T	2.00
5	67	M	Headache	L T	4.00
6	66	F	Haemorrhage	L T	1.50
7	39	M	Headache	R F	2.00
8	50	M	Haemorrhage	R P/T	3.62

* R: right; L: left; F: frontal; T: temporal; P: parietal; O: occipital.

AVM: arteriovenous malformations.

### Immunohistochemistry

Human brain specimens were embedded in paraffin and cut into 6-μm sections, which were deparaffinized with xylene and rehydrated with ethanol, following antigen retrieval with antigen unmasking solution (Vector Laboratories, Burlingame, CA, USA) according to the manufacturer's instructions. Endogenous peroxidase activity was blocked by incubation in 1% H_2_O_2_ at room temperature for 30 min. After several washes with PBS, sections were incubated in blocking solution (2% goat serum, 0.3% Triton X-100 and 0.1% bovine serum albumin in PBS) for 1 hr at room temperature. Primary antibody rabbit polyclonal anti-Notch4 (1:500; Sigma-Aldrich, St. Louis, MO, USA) or rabbit anti-Notch4 (1:100; Millipore, Billerica, MA, USA) was added to blocking buffer and incubated with sections at 4°C overnight. Sections were then washed with PBS and incubated with biotinylated goat anti-rabbit (1:200; Santa Cruz Biotechnology, Santa Cruz, CA, USA) for 1 hr at room temperature. Avidin–biotin complex (Vector Elite; Vector Laboratories) and a diaminobenzidine or nickel solution (Vector Laboratories) were used to obtain a visible reaction product. The slide examiners were blinded to the source of the specimen (brain AVMs *versus* control). A Nikon microscope and a Nikon digital colour camera were used for examination and photography of the slides, respectively.

### Double immunostaining

Double immunostaining was performed on brain sections as previously described 12. The primary antibodies used, in addition to Notch4 antibody, included mouse anti-CD31 (PECAM1; Dakocytomation, Denmark; 1:100) and rabbit anti-smooth muscle α-actin (α-SMC; 1:300; Maine Biotechnology Service, Portland, ME, USA). FITC-labelled *Lycopersicon esculentum* (tomato) lectin (1:300; Vector Laboratories, Burlingame, CA, USA) was also used for vessel staining. The secondary antibodies were Alexa Fluor 488-, 594-, or 647-conjugated donkey anti-mouse or anti-rabbit IgG (1:200–500; Molecular Probes, Grand Island, NY, USA). Slides were mounted with proLong Gold antifade reagent with DAPI (Molecular Probes). Fluorescence signals were detected with an LSM 510 NLO Confocal Scanning System mounted on an Axiovert 200 inverted microscope equipped with a two-photon Chameleon laser. Selected images were viewed at high magnification, and 3-dimensional images were constructed using IMARIS software. Controls included omitting either the primary or secondary antibody or preabsorbing the primary antibody.

### Western blotting

Tissues were dissected from frozen brains. Protein was isolated and Western blotting was performed as previously described 12. The primary antibodies were affinity-purified rabbit polyclonal anti-Notch4 (1:1000; Millipore) and mouse monoclonal anti-actin (1:20,000; Oncogene Science, Cambridge, MA, USA). Membranes were washed with PBS/0.1% Tween 20, incubated at room temperature for 60 min. with horseradish peroxidase-conjugated anti-mouse or anti-rabbit secondary antibody (1:3000; Santa Cruz Biotechnology), and washed three times for 15 min. Peroxidase activity was visualized by chemiluminescence. Notch4 antibody was removed with stripping buffer at 50°C for 30 min., followed by washing with PBS/Tween-20, and membranes were re-probed with anti-actin.

## Results

**Figure 1:**
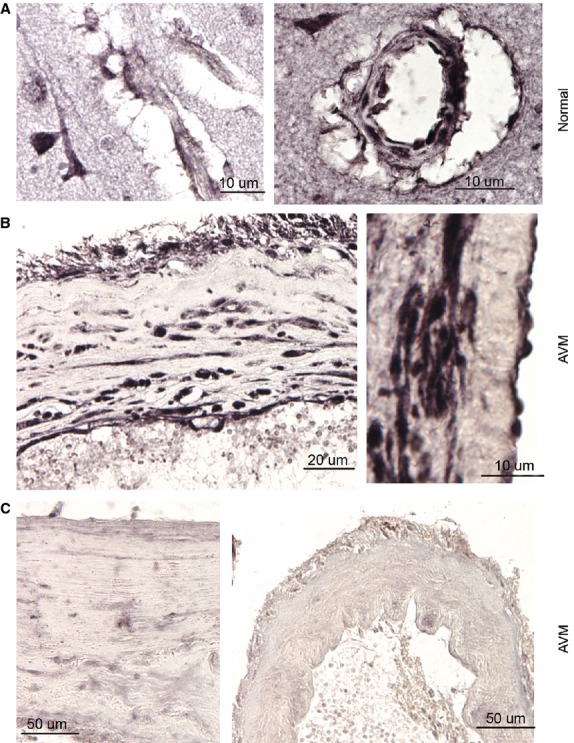
Up-regulation of Notch4 in the normal brain vascular tissue and brain arteriovenous malformations (AVMs) in human. (A) Notch4 expression was observed in capillary (left panel) and small artery (right panel) in normal human brain. (B) Notch4 was highly expressed in the cells of abnormal vascular wall in brain AVMs. Left panel, low-magnification; Right panel: high-magnification. (C) Notch4 was slightly (left panel) or barely (right panel) expressed in cells of selected abnormal vascular walls in brain AVMs.

**Figure 2:**
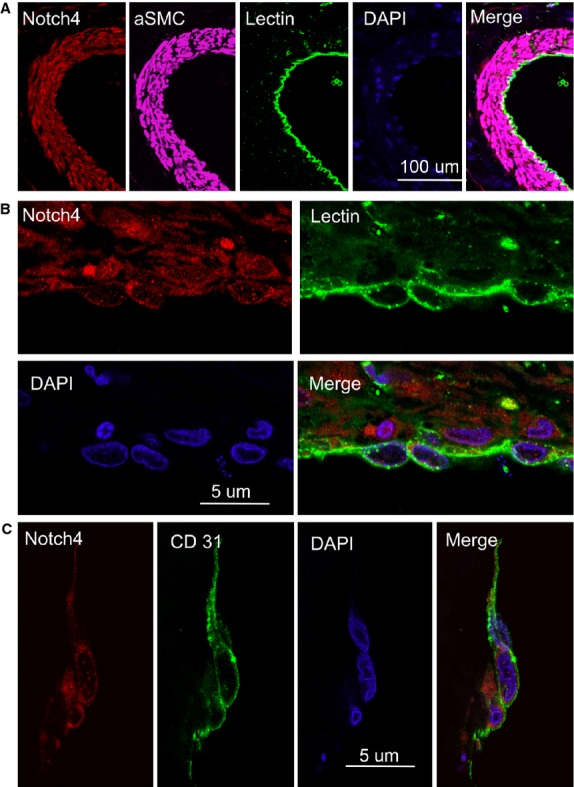
Expression of Notch4 in the endothelial cells in human brain arteriovenous malformations (AVMs). (A) Double immunostaining of human brain AVM (low-magnification) shows that Notch4 (red) was expressed in lectin-positive cells (green), and SMC-positive cells (purple). DAPI (blue) was used to counterstain nuclei. (B) Double immunostaining of human brain AVM (high-magnification) shows cytoplasmic Notch4 (red) and cytoplasmic CD31 (green) in the same (vascular endothelial) cells. DAPI (blue) was used to counterstain nuclei. (C) Double immunostaining of human brain AVM shows that Notch4 (red) was expressed in cytoplasmic CD31-positive cells (green). DAPI (blue) was used to counterstain nuclei.

**Figure 3:**
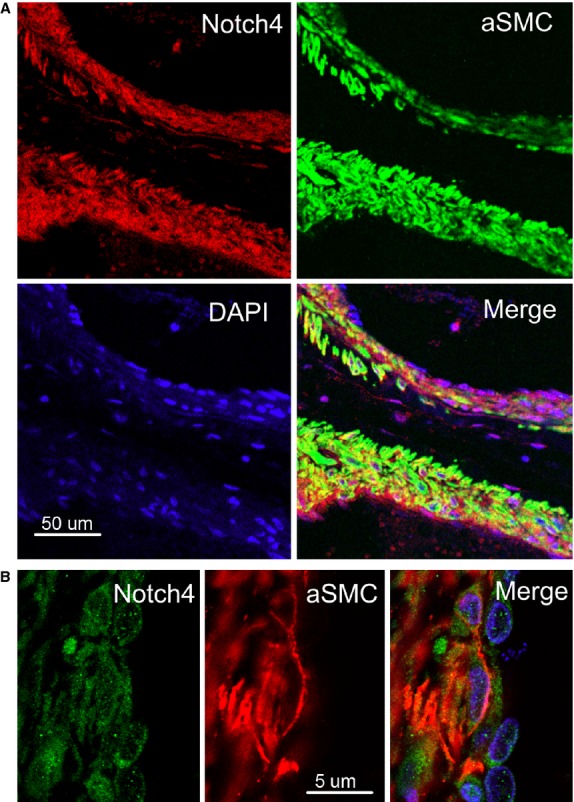
Expression of Notch4 in the smooth muscle cells (SMC) in human brain arteriovenous malformations (AVMs). (A) Double immunostaining of human brain AVM (low-magnification) shows that Notch4 (red) was expressed in α-SMC-positive cells (green). DAPI (blue) was used to counterstain nuclei. (B) Double immunostaining of human brain AVM (high-magnification) shows Notch4 (green) and α-SMC (red) in the same (vascular smooth muscle) cells. DAPI (blue) was used to counterstain nuclei.

To investigate the expression profile of Notch4 in human brain AVMs, immunostaining was first performed on the normal human brain sections using antibody against Notch4. As shown in Figure 1, Notch4 was expressed not only neuronal cells, but also weakly expressed in the cells of capillary and small artery in normal human cortex. We also found that Notch4 expression was significantly increased in the cells of some abnormal vessel walls of brain AVMs, suggesting that Notch4 was selectively expressed in the subset of brain AVM nidus. To determine the phenotype of Notch4-expressing cells, double immunostaining was performed using anti-α-SMA to label SMCs and anti-CD31 or lectin for endothelial cells. The results were recorded with two-photon confocal microscope. As shown in Figures 2 and 3, Notch4-positive cells expressed CD31 and lectin as well as α-SMC, suggesting that Notch4 was expressed in both endothelial cells and SMCs in brain AVMs. High magnification view indicates that Notch4 was mainly expressed in the cytoplasm of both endothelial cells and SMCs (Figs 2 and 3), when anti-Notch4 activating antibody was used.

**Figure 4:**
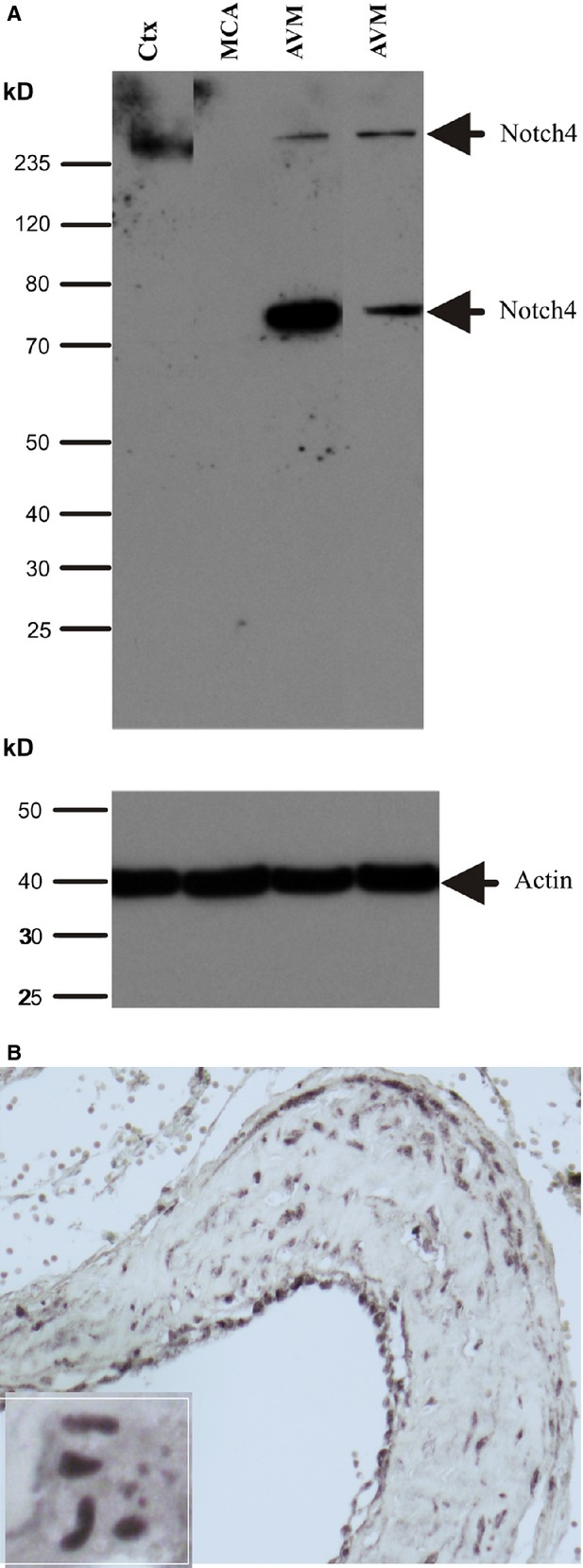
Activation of Notch4 in human brain arteriovenous malformations (AVMs). (A) Western blot was performed using an antibody generated from a peptide against Notch4-activated form. A fragment of activated Notch4 was observed in brain AVM, but barely expressed in normal control human brain. Ctx, cortex; MCA, middle cerebral artery. (B) Immunostaining was performed using the same antibody described above and Notch4-activated positive staining was found in the endothelial cells and smooth muscle cells. Notch4-activated form was mainly expressed in the nuclei (insert).

By sequence similarity, the Notch intracellular domain (NICD) is contained within amino acids 1467–2003. Anti-Noth4 antibody from Millipore used here was generated using the peptide corresponding to amino acids 1796–1964 of Notch4, which is able to detect Notch4-activated form. To extend these immunohistochemical studies and determine whether Notch4 was activated in brain AVMs, Western blot analysis was performed. As shown in Figure 4, a band of the predicted size (56 kD, cleaved form of Notch4) was detected in patients with brain AVM, irrespective of clinical presentation, but not in the normal cortex and normal human middle cerebral artery (MCA), which is comparable in calibre to AVM vessels. The intensity of the Notch4 signalling varied across patients, which might reflect different stages of patients with brain AVMs. In agreement with data obtained by Western blot, immunostaining with a Notch4-activating rabbit antibody confirmed that Notch4 protein was expressed in the nuclei of endothelial cells and SMCs, suggesting that Notch4 signalling was activated in these cells in brain AVMs (Fig. 4B).

## Discussion

In this study, we found that Notch4 was predominantly expressed in the endothelial cells of normal adult human brain, and was increased not only in endothelial but also in SMCs of human brain AVMs. We also found that Notch4 was activated in endothelial and SMCs of human brain AVMs. Our data suggest that activation of Notch, including Notch4, signalling may contribute to vascular malformations not only in mice during development but also in adult brain in human.

Notch4 is synthesized in the endoplasmic reticulum as an inactive form. It functions as a receptor for transmembrane ligands. Upon ligand binding, Notch4 undergoes sequential proteolytic processing to release the intracellular domain (NICD), which translocates to the nucleus, where it forms a transcriptional activation complex. The nuclear localization of an intracellular domain of Notch4 is the feature of active Notch signalling. Our data show that Notch4 protein is located in the nuclei of endothelial and SMCs of human brain AVMs by analysis of immunocytochemistry. Activated form of Notch4 is also detected in surgical specimens of brain AVMS by Western blot, suggesting that Notch4 signalling is activated in human brain AVMs. Although the activation of Notch4 signalling in surgical specimens cannot state that this pathway directly causes the pathogenesis of brain AVMs in human, it suggests that this signalling is involved at some stage in the development of brain AVMs in human, based on following findings: (1) Expression of Notch4 is restricted to developing arteries 13. Expression of activated Notch4 or Jagged1 can promote the differentiation of cultured endothelial cells into vessel-like structures 14. Directed expression of a constitutively active form of Notch4 within mouse endothelial cells produces profound blood vessel enlargement and AV shunting, which are hallmarks of AVM, and leads to embryonic lethality 9, indicating that the vascular development depends on Notch signalling. (2) The inducible expression of an activated *Notch4* transgene in adult mice causes AVMs, along with vessel arterialization, such as the induction of venous expression of the ephrin B2 gene. These malformations are reversible if activated *Notch4* transgene expression is inhibited 9, demonstrating that the ability of Notch4 signalling to arterialize blood vessels is not confined to the embryonic period. (3) Hes1, downstream target of Notch signalling, is activated in brain AVMs in human 6–11. In this study, we find that Notch4 is activated in the endothelial and SMCs in surgical specimens of brain AVMS, indicating the importance of these molecules in pathogenesis of brain AVMs in human.

In addition, a number of other factors involved in vascular development are expressed in the vascular cells of AVMs. VEGF, a promoting angiogenesis factor, is highly expressed in brain AVMs 15. VEGF receptors Flik-1 and Flt-1 are significantly greater in brain AVMs than in the control brain tissue samples 16. Hypoxia-inducible factor 1, alpha subunit (HIF-1a), a VEGF upstream factor, is induced in the endothelial cells of brain AVMs 15–17. Delta-like 4 (Dll4), a transmembrane ligand for Notch receptors, has emerged as the critical ligand in Notch signalling-mediated vascular malformations in mice. Genetic deletion of Dll4 in mice produces profound vascular defects 3–18. Heterozygous deletion of *dll4* exhibits a striking vascular phenotype, with greatly increased numbers of filopodia-extending endothelial tip cells. Filopodia extension in *dll4−/−* retinal vessels requires VEGF and is inhibited when VEGF signalling is blocked 19. Another study also shows that blocking VEGF signalling with a small molecule inhibitor prevents both normal endothelial sprouting and the ectopic sprouting observed in *dll4* morphant embryos 20. These data suggest that Notch and VEGF signalling is essential for proper vascular morphogenesis.

In addition to VEGF, abnormal expression patterns of angiopoietin-2 (Ang-2), tie-2 and MMP-9 are observed in surgical specimens of brain AVMs 21. MMP-9 is a key downstream consequence of VEGF and Ang-2 activity, contributing to the angiogenic phenotype 21, whereas Ang-2/Tie pathway is also associated with Notch signalling 22 Taken together, the action of these factors alone is not sufficient to explain the complex patterning of the brain AVMS; many factors or pathways may be involved in the development of vascular malformations. Considering the role of Notch signalling in vascular development and activation of Notch signalling in brain AVMs, the proper activation of Notch signalling may play a critical role in the pathogenesis of brain AVMs in human.
